# Investigating the Neural Mechanisms of Self-Controlled and Externally Controlled Movement with a Flexible Exoskeleton Using EEG Source Localization

**DOI:** 10.3390/s25113527

**Published:** 2025-06-03

**Authors:** Takayuki Kodama, Masahiro Yoshikawa, Kosuke Minamii, Kazuhei Nishimoto, Sayuna Kadowaki, Yuuki Inoue, Hiroki Ito, Hayato Shigeto, Kohei Okuyama, Kouta Maeda, Osamu Katayama, Shin Murata, Kiichiro Morita

**Affiliations:** 1 Department of Physical Therapy, Faculty of Health Sciences, Kyoto Tachibana University, 34 Oyake yamada, Yamashina-ku, Kyoto-City 607-8175, Kyoto, Japan; nishimoto-ka@tachibana-u.ac.jp (K.N.); shigeto@tachibana-u.ac.jp (H.S.); murata-s@tachibana-u.ac.jp (S.M.); 2 Graduate School of Robotics and Design, Osaka Institute of Technology, 1-45 Chayamachi, Kita-ku, Osaka-City 530-8568, Osaka, Japan; masahiro.yoshikawa@oit.ac.jp (M.Y.);; 3 National Center for Geriatrics and Gerontology, Center for Gerontology and Social Science, 7-430 Morioka-cho, Obu-City 474-8511, Aichi, Japan; katayama.o@ncgg.go.jp; 4 Cognitive and Molecular Research Institute of Brain Diseases, Kurume University, Kurume-City 830-0011, Fukuoka, Japan; kiichiro@kurume-u.ac.jp

**Keywords:** self-controlled motor tasks, externally controlled tasks, motor imagery, EEG, ERD/ERS, eLORETA, flexEXO, sensorimotor cortex, dACC, DLPFC, VLPFC, grasp, neurorehabilitation, assistive devices, brain–computer interfaces, motor learning, neural plasticity

## Abstract

**Highlights:**

**What are the main findings?**

- Self-controlled tasks with flexEXO enhance activation in motor-related brain areas.
- Externally controlled tasks activate sensory feedback and error-monitoring regions.

**What is the implication of the main finding?**

- Self-controlled movement may better support motor learning and neurorehabilitation.
- flexEXO enables active engagement in motor training for users with limited hand function.

**Abstract:**

Background: Self-controlled motor imagery combined with assistive devices is promising for enhancing neurorehabilitation. This study developed a soft, Flexible Exoskeleton (flexEXO) for finger movements and investigated whether self-controlled motor tasks facilitate stronger cortical activation than externally controlled conditions. Methods: Twenty-one healthy participants performed grasping tasks under four conditions: Self-Controlled Motion (SCC), Other-Controlled Motion (OCC), Self-Controlled Imagery Only (SCIOC), and Other-Controlled Imagery Only (OCIOC). EEG data were recorded, focusing on event-related desynchronization (ERD) in the μ and β bands during imagery and motion and event-related synchronization (ERS) in the β band during feedback. Source localization was performed using eLORETA. Results: Higher μERD and βERD were observed during self-controlled tasks, particularly in the primary motor cortex and supplementary motor area. Externally controlled tasks showed enhanced activation in the inferior parietal lobule and secondary somatosensory cortex. βERS did not differ significantly across conditions. Source localization revealed that self-controlled tasks engaged motor planning and error-monitoring regions more robustly. Conclusions: The flexEXO device and the comparison of brain activity under different conditions provide insights into the neural mechanisms of motor control and have implications for neurorehabilitation.

## 1. Introduction

Motor imagery (MI) is the mental reproduction of visual and motor sensory information without actual physical movement [1,2]. In recent years, MI has been reported to effectively promote brain activity and assist motor learning in rehabilitation and sports science [3,4,5], and its range of applications is expanding. It is known that MI activates brain processes involved in planning and preparing for movement, and it can induce neuronal activity in motor-related areas even without actual movement [6,7]. Studies have shown that brain activity during MI and motor execution (ME) share many similarities. In particular, motor-related areas such as the primary motor cortex (M1), supplementary motor area (SMA), and premotor cortex (PMC) are commonly activated [8,9].

However, certain issues need to be addressed to maximize the benefits of MI. When using MI in rehabilitation, there are differences in brain nerve activity between the Self-Controlled Condition (SCC), in which active physical manipulation is involved, and the Other-Controlled Condition (OCC), in which brain representations of the movement are induced. For example, in self-controlled movements, the brain compares and checks the actual movement results against the predictions. This process is thought to increase the activation of the primary motor cortex and supplementary motor area, leading to motor learning and the restructuring of sensorimotor functions [10,11,12]. It is thought that self-regulatory movements, in which the entire process from planning to execution is processed consistently in the brain, promote more effective neuroplastic changes [13,14]. In other words, it is believed that consistently simulating and executing the entire process from MI to ME in the brain, rather than simply recalling the movement, effectively activates motor-related brain areas. However, there is a difference between the patient’s motor prediction and ME in approaches that focus on other-controlled movements. Because motor feedback is not fully utilized, it has been pointed out that there is a limit to the effectiveness of functional improvement, even when MI is used [15,16]. It has been shown that active self-controlled movements significantly increase neural activity in the primary motor cortex and supplementary motor area compared with passive externally controlled movements [17,18]. In particular, in patients with sensorimotor dysfunction, such as stroke or traumatic brain injury, voluntary motor control is impaired, and passive intervention approaches, which can be thought of as ‘other-controlled’ movements, often do not lead to sufficient functional recovery [19,20]. Based on the above findings, it can be concluded that incorporating self-regulatory elements into rehabilitation using MI is essential. Recent studies have further investigated the neural mechanisms underlying the differences between self-controlled and externally controlled movements. For instance, Derosiere et al. used functional magnetic resonance imaging (fMRI) to demonstrate that self-controlled movements engage a wider network of motor-related areas, including the primary motor cortex, supplementary motor area, and premotor cortex, compared to externally controlled movements [21]. These findings suggest that self-controlled movements promote more effective sensorimotor integration and motor learning. Additionally, the use of motor imagery in rehabilitation has been shown to enhance neural plasticity and improve motor function in patients with neurological disorders [22,23]. Advanced assistive devices, such as exoskeleton robots, have the potential to facilitate self-controlled movements and provide appropriate sensory feedback, as demonstrated by Ramos-Murguialday et al. in a study using a brain–machine interface system to control an exoskeleton robot for stroke rehabilitation [24].

Self-regulatory MI is expected to promote the restructuring of functional neural networks by effectively activating the brain processes involved in actual motor planning and execution rather than merely recalling movements [25]. In addition, it is thought that consistency between motor prediction and execution is important for the effective use of self-regulatory MI. It has been shown that the consistency between motor prediction and actual sensory feedback improves the accuracy of motor control [26], and it has been suggested that the sense of agency, which is an element that affects the recovery of motor function, depends on the consistency between motor prediction and execution [27]. Furthermore, MI supports the function of forward model encoding that precedes motor execution, and comparisons of the executed movement with the image (comparator model) promote the reconstruction of sensorimotor information in the brain [28,29]. In other words, it is thought that the synchronization of motor imagery and actual movement strengthens the matching of predictions and sensory feedback in the brain’s information-processing system, contributing to functional recovery and learning promotion. This congruence between motor prediction and execution is expected to be particularly prominent in self-regulatory MI [30,31]. These findings suggest that congruence between motor prediction and execution is an important factor in enhancing the effects of self-regulatory MI and highlights the importance of devising an approach that simultaneously utilizes self-regulation and motor imagery. Despite the growing interest in the use of motor imagery and assistive devices for neurorehabilitation, previous studies have several limitations. First, there is a lack of direct comparisons between self-controlled and externally controlled movements, which limits our understanding of the specific neural mechanisms involved [21]. Second, most assistive devices for hand function are rigid and do not allow for natural, complex movements [32]. Finally, the neural correlates of the effects of self-controlled motor tasks on brain activity remain poorly understood [33]. The current study aims to address these limitations by developing a novel soft robotic exoskeleton and investigating the differences in brain activity patterns under self-controlled and externally controlled conditions.

The sense of agency and contingency between motor prediction and execution is related to neurophysiological indicators of brain activity, such as event-related desynchronization (ERD) and event-related synchronization (ERS). The sense of agency refers to the ability to perceive the causal relationship between a specific action and its result and involves the recognition that one’s actions cause specific results. If this sense of agency is strongly perceived, the effect of self-controlled MI may be further enhanced. For example, when motor prediction and sensory feedback are in agreement, this strengthens the association and promotes efficient information processing and learning in the brain. The process of matching predictions and sensory feedback can also be captured by ERD and ERS EEG indicators (particularly in the mu and beta wavebands) and has been used in many studies to evaluate cortical activity during motor imagery and motor execution [34,35]. However, although it has been reported that the activity of motor-related areas, such as M1 and SMA, increases during self-initiated movement [36], the functional differences that exist in self-controlled active movement that enhance the congruence of MI and ME when compared to external control by another person have not been verified, nor the effects they have on ERD and ERS. The significance of verifying these points lies in the fact that, in rehabilitation aimed at reorganizing brain function, it is important to maximize active elements and create an environment in which patients can predict, select, and execute movements. Conversely, in patients with severe motor dysfunction, it can be difficult for them to perform self-regulated movements. For such patients, it is essential to introduce external support that can appropriately assist movement intention and effort and enable smooth movement execution [37,38]. For example, in rehabilitation using robotic technology, it is possible to detect a patient’s motor intentions and provide appropriate assistance to encourage the emergence of self-regulated movements [39]. With this approach, it is hoped that effective rehabilitation using MI will be possible, even for patients with severe motor dysfunction. However, for patients with severely reduced voluntary control, the problem is that it is difficult for them to perform self-regulated movements in the first place. For this reason, there is increasing significance in developing a system that provides the necessary assistive force in line with the timing and intention of the movement that the patient wants to perform by combining an assistive device that supplements motor function.

In recent years, research has progressed on lightweight device materials that consider the comfort of wearing them, and the potential of technology that assists with the voluntary movement of the lower limbs and hand/upper limb functions, such as grasping and reaching, has been suggested [40,41]. The introduction of this type of assistive device is expected to enable patients to synchronize the motor images in their brains with the actual device operation and reproduce self-regulatory elements. We believe that the soft exoskeleton used in this study has the potential to support the realization of self-regulatory MI training by accurately assisting the necessary parts while minimizing skin contact and maximizing the use of the patient’s remaining functions. In particular, in the rehabilitation of motor dysfunction in the fingers, the reacquisition of grasping movements is one of the important goals, and the use of a soft exoskeleton for the fingers is seen as a promising approach [42].

In this study, we developed and produced a dedicated finger exercise assist device, the Flexible Exoskeleton (flexEXO), which can also be used by patients with reduced voluntary finger movement. We aimed to verify the effects of self-controlled operation on brain activity using neurophysiological methods. In particular, we compared brain activity under self-controlled and other-controlled conditions and clarified the neurophysiological effects using ERD and ERS indices. flexEXO is designed using lightweight flexible materials and can provide appropriate assistive force while tracking complex movements. It was hoped that this device would enable patients with severe motor function disorders to perform self-controlled MI training. The results of this research have the potential to contribute to the development of rehabilitation aimed at reorganizing sensorimotor function in the future. We aim to help increase the functional independence of patients with sensorimotor dysfunction. The rehabilitation approach that combines MI and assistive devices is expected to bring new hope to many patients suffering from the after-effects of stroke, traumatic brain injury, and other conditions. The flexEXO is expected to provide a new option for rehabilitation aimed at restoring hand movement function and contribute to improving the quality of life of patients.

The main contributions of this study are as follows:

1. A comprehensive review of the neural mechanisms underlying the differences between self-controlled and externally controlled movements, highlighting the potential benefits of self-controlled movements for motor learning and neurorehabilitation.
2. An analysis of the role of motor imagery in enhancing neural plasticity and motor function in patients with neurological disorders, emphasizing the need for advanced assistive devices to optimize the benefits of motor imagery training.
3. A discussion of the potential of advanced assistive devices, such as exoskeleton robots, in facilitating self-controlled movements and providing appropriate sensory feedback for neurorehabilitation.

## 2. Materials and Methods

### 2.1. Participants

The participants were recruited from young individuals living in Yamashina Ward, and the experiment was conducted on 25 healthy males (22.8 ± 2.1 years old). In this initial investigation, we focused on male participants to control for potential confounding factors related to gender differences in motor control and brain activity patterns. Previous research has shown that hormonal fluctuations in women can lead to greater individual variability compared to men [43]. By including only male participants, we aimed to minimize this variability and establish a baseline for the neural mechanisms underlying self-controlled and externally controlled movements using flexEXO. The exclusion criteria were a history of neurological or psychiatric disorders, use of drugs affecting the central nervous system, and physical or cognitive limitations that would prevent participants from completing the experimental tasks. All participants were fully informed of the purpose, content, and procedures of this study, both verbally and in writing, and their consent was obtained. The participants’ attributes are presented in Table 1.

### 2.2. Method

#### 2.2.1. Experimental Procedure

**Figure 1:**
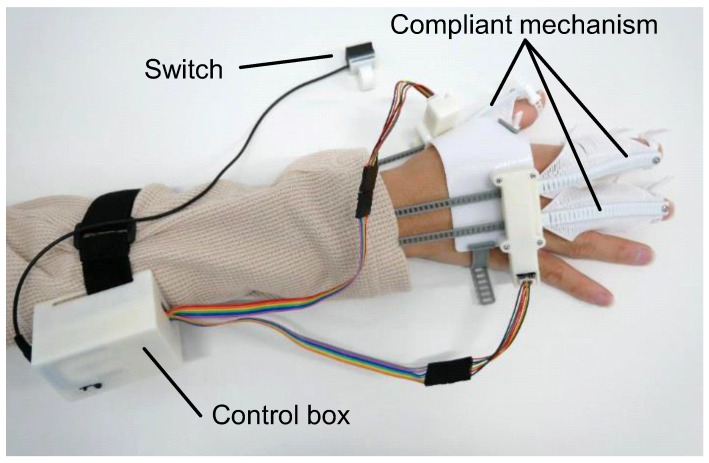
Configuration of flexEXO.

In this experiment, we examined how neural activity in the brain during the MI of finger movements relates to neural activity during grasping movements, whether controlled by oneself or another person. As part of the experimental procedure, we first conducted an evaluation of item 5Vd, ‘Thumb-tip’, from the Kinesthetic and Visual Imagery Questionnaire-20 (KVIQ) [44], which assesses kinesthetic and visual imagery. The participants were then asked to grasp an object in front of them with their right hand while wearing flexEXO (Figure 1). The object used for grasping was an aluminum alloy cylinder, with a diameter of 4 cm, a length of 7 cm, and a weight of 190 g, which was calculated as the average size of objects used in previous grasping experiments [45,46]. The position of the object to be grasped was adjusted to the midline of the participant’s body, with the right elbow resting on the desk and the right forearm lying comfortably on the desk in a seated position after adjusting the chair height, considering the variation in upper limb and trunk lengths among participants. The starting position of the right hand was adjusted to wrap around the object being grasped, with a starting angle of 0° at the metacarpophalangeal joint from the index finger to the little finger. Four grasping task conditions were set, and electroencephalography was performed to verify neural activity in the brain during each condition.

#### 2.2.2. flexEXO

**Figure 2:**
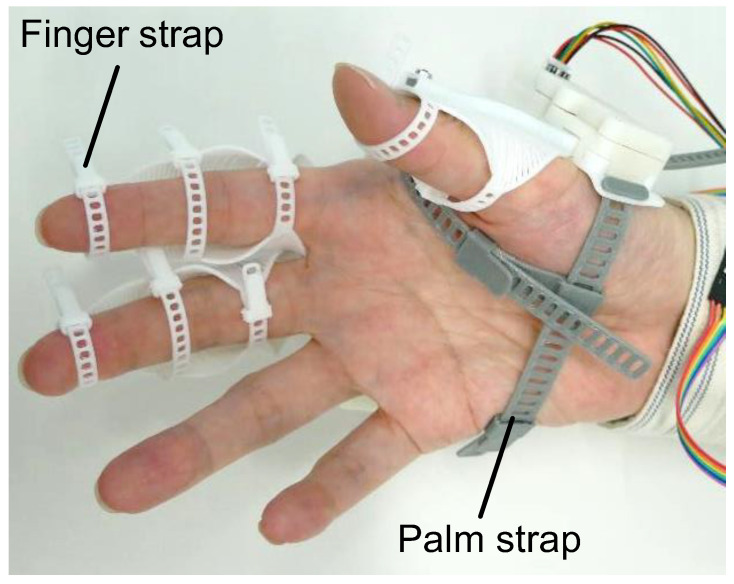
Straps for wearing exoskeletons.

The configuration of flexEXO is illustrated in Figure 1. It consisted of three compliant mechanisms worn on the hand, a control box worn on the arm, and a push switch for controlling the compliant mechanisms. The control box contained a micro-controller (Arduino Nano Every) (manufacturer: Arduino AG, Milan, Italy), a motor driver (DRV8833 motor driver) (Pololu Corporation, Las Vegas, NV, USA), and a 9V rechargeable battery. flexEXO weighed 177 g (the part attached to the hand weighed 73 g). flexEXO could be adjusted by tightening the finger and palm straps, as shown in Figure 2.

**Figure 3:**
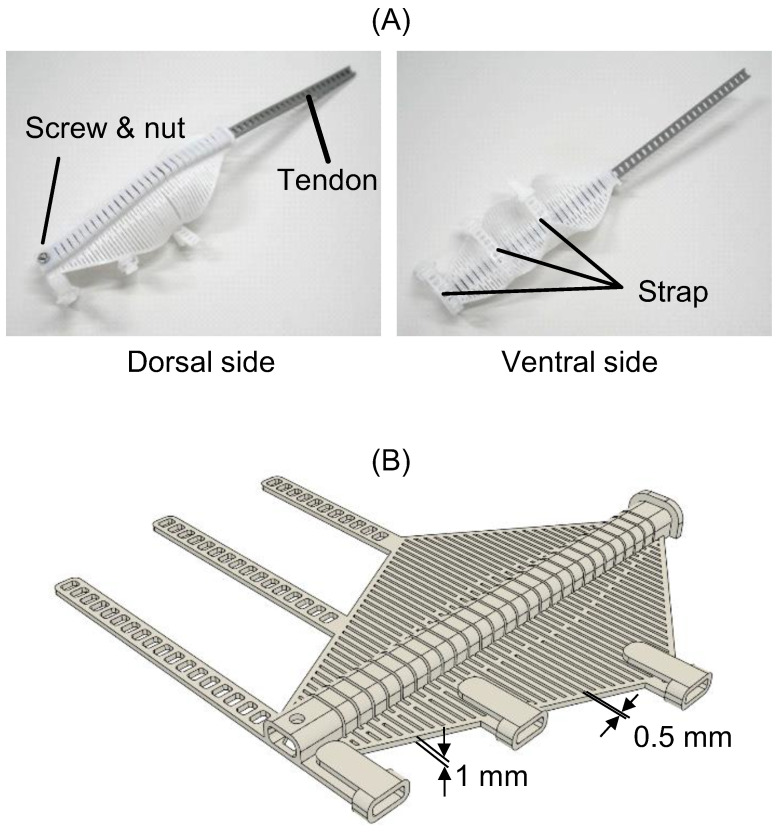
TPU 3D-printed compliant finger mechanism: integrated model of tendon-driven slit structure and flat CAD design. (**A**) Compliant mechanism and tendon for the index finger. (**B**) CAD model of the compliant mechanism.

The compliant mechanism for the index finger is shown in Figure 3A. The tendon passed through a center tunnel containing multiple slits. The tendon end and the tip of the mechanism were fixed with a screw and nut. The ventral side was open to allow for tactile sensation and natural finger friction. The compliant mechanism and tendon were 3D-printed using thermoplastic polyurethane (TPU) resin (TPU95A, eSUN Industrial Co., Ltd., Shenzhen, China) on a 3D printer (Ender-3 S1, Creality 3D Technology Co., Ltd., Shenzhen, China). Figure 3B displays the CAD model of the compliant mechanism. The mechanism with the straps was 3D-printed in a flat state. It was designed based on the average dimensions of an adult male hand.

**Figure 4:**
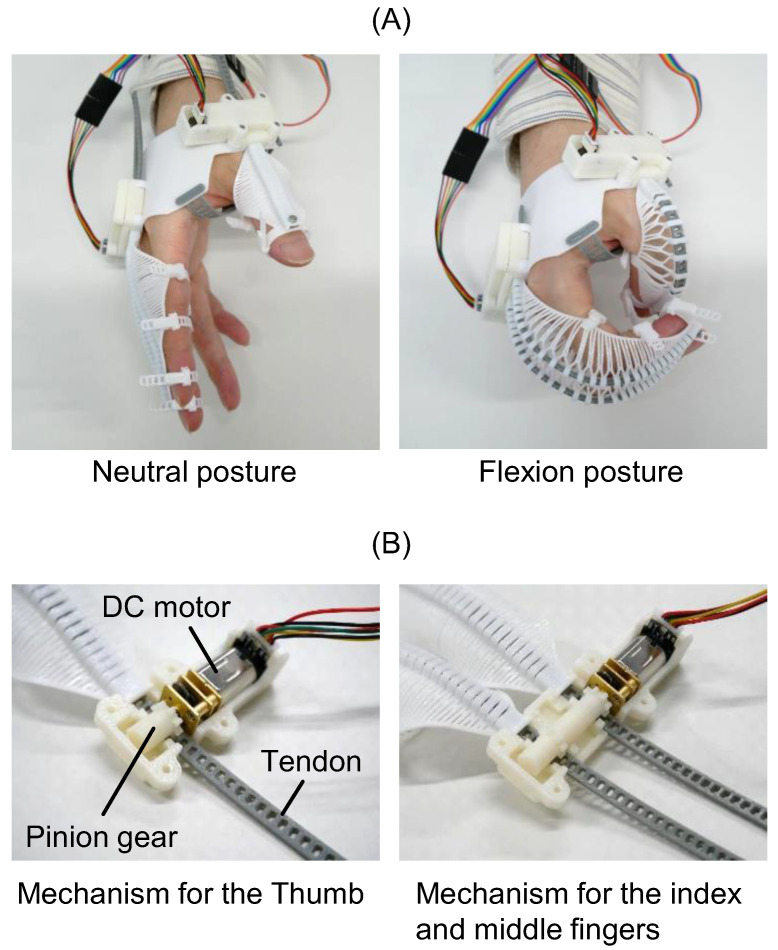
flexEXO finger actuation system: tendon slit flexion and pinion gear transmission. (**A**) Flexion of flexEXO. (**B**) Mechanisms for driving tendons.

flexEXO was driven by tendons. When the tendon was pushed towards the tip of the compliant mechanism, it flexed while spreading at the slits, as shown in Figure 4A. When the tendon was pulled back, the compliant mechanism was extended. flexEXO was driven by two DC (direct current) motors (HPCB 6V Micro Metal Gearmotor 250:1, Pololu Corporation, Las Vegas, NV, USA). Figure 4B shows the configuration of the mechanism that transmitted power from the motors to the tendons. The tendon had multiple slots meshed with a pinion gear attached to a DC motor. The rotational motion of the DC motor was converted into linear tendon motion. The tendons for the index and middle fingers were driven simultaneously by two pinion gears connected to a single DC motor. A magnetic encoder (Pololu Corporation, Las Vegas, NV, USA) was attached to the DC motor to restrict the range of motion. The pinion gears and cases were 3D-printed using acrylonitrile butadiene styrene (ABS) resin on a 3D printer (F170, Stratasys Ltd., Eden Prairie, MN, USA).

The wearer controlled flexEXO by pressing a switch with their healthy finger. This switch, which our research group developed in the past [47], detected the pressure ap-plied based on a phototransistor (SG-105, KODENSHI Corp., Kyoto, Japan). Each time the switch was pressed, flexEXO alternated between flexion and extension. If the switch was held down, flexEXO continued to move. This functionality allowed the wearer to adjust the degree of flexion based on the object they were grasping. The command cycle time for the motor was 10 milliseconds, and transitioning from a neutral posture to a fully flexed position took 1.9 s. The movement stopped once the number of motor revolutions reached a specified threshold to prevent excessive flexion. Please refer to reference [48] for the range of motion and grasping force when wearing flexEXO.

#### 2.2.3. Experimental Conditions

This section describes the six experimental conditions used in our study: the Rest Condition (RC), the Self-Controlled Condition (SCC), the Other-Controlled Condition (OCC), the Self-Controlled Imagery-Only Condition (SCIOC), the Other-Controlled Imagery-Only Condition (OCIOC), and the Motion-Only Condition (MOC). Each condition served a specific purpose in investigating the neural mechanisms underlying self-controlled and externally controlled movements using flexEXO.

1. Rest Condition (RC)

Before the grasping experiment, the participants were asked to sit in a relaxed chair position, and their EEG signals were measured for 2 min. During this time, they were instructed to gaze absentmindedly at the desk in front of them. The participants were instructed to keep their bodies still and minimize eye movements while maintaining a relaxed state. EEG data from the resting condition were used as the baseline for each participant and were necessary for calculating the ERD during the grasping condition [35]. EEG data from the resting condition also served as a baseline for each participant’s state of arousal and played an important role in evaluating changes in brain activity during the task [49].

2. Self-Controlled Condition (SCC)

After confirming that the participants were ready to begin the experiment in a seated position, they were asked to rest for 10 s. Subsequently, at the start of the beep sound (2 kHz), the participant was asked to imagine in their mind’s eye (Imagery Phase [IP]) that they were grasping the object by flexing their fingers. At the end of the IP, when they were asked to imagine the grasping motion again, they pressed the start button on the device with their left hand, which was on the opposite side of their body, causing their fingers to flex and assume the position of grasping the object. Because the active image of the user and the actual grasping movement occured synchronously, self-control was activated, enabling the brain to compare and collate predictions of movement with motor execution. The device takes 1.8 s to move from the start to the grasping hand shape; therefore, this movement phase (Motion Phase: MoP) was set to 2 s. Subsequently, the Feedback Phase (FBP), during which the participant was instructed to reflect on the consistency of the results of the movement while holding the object, was set for 2 s. No finger movements occurred during the Feedback Phase. The above three phases, IP, MoP and FBP, were performed as a set, and all were performed with eyes open. The 10 s resting period at the start was also set as the interval time between sets. For the IP, the instruction was, ‘Please imagine that you are grasping the tube in front of you with your right fingers once within the given 2 s.’ The participants were instructed to press the button on the device when they had completed the image. Since it was expected that the timing of pressing the button would differ between participants, we explained in advance that the time range for the IP was approximately 2 s and conducted a rehearsal in which they performed the MI by grasping for 2 s and then pressed the button. The rehearsal was carried out only once, without the device being worn or the object being presented, to exclude any learning effects. The time setting for the IP was set to 2 s because it is thought that the MI activated prior to the execution of the movement is equivalent to the actual movement [50,51]. After the experiment, the time from the start to the point at which the button was pressed was calculated, and all participants were asked whether they were able to follow the instructions within 2 s. During the 10 s interval, a blackout curtain was placed in front of the face to block visual information related to the hands. Simultaneously, the device in the flexed position was reset to the starting position by the experimenter. After this interval, a beep sound was heard again, and the next set began.

3. Other-Controlled Condition (OCC)

The OCC was set up in the same manner as the SCC for the experimental phase, with one set consisting of 16 s of IP, MoP, FBP, and intervals. The only difference from the SCC was that, after 2 s of IP, the experimenter pressed a button to drive the device, as in other-controlled imagery. The contents of the other phases were the same as those in the SCC.

4. Self-Controlled Imagery-Only Condition (SCIOC)

The SCIOC condition was established to compare and verify the brain’s functional state of MI without ME under self-control, in comparison with the SCC and OCC conditions. Under these conditions, the device was worn, and the same IP, MoP, FBP, and intervals as in the SCC were configured as one set of 16 s. The MoP content differed from that of the SCC in that, after the IP, the participant pressed the button, but the device was not triggered. The contents of the other phases were the same as those in the SCC. In addition, the time taken to press the button was measured and confirmed in the same manner as in the SCC.

5. Other-Controlled Imagery-Only Condition (OCIOC)

Similarly to SCIOC, OCIOC was also set as a condition to compare and verify the brain’s functional state during MI without ME under the control of others, in comparison with SCC and OCC. In this condition, with the device attached, the 16 s interval of IP, MoP, FBP, and interval was configured as one set, the same as in the OCC. The MoP content differed from that of the OCC in that the experimenter pressed the button after a 2 s interval of IP, but the device was not triggered. The contents of the other phases were the same as those in the OCC.

6. Motion-Only Condition (MOC)

The MOC is a basic condition that isolates brain activity related to the ‘button-pressing’ movement observed in the SCC and SCIOC. By subtracting the brain activity data of the IP and MoP phases from those of the SCC and IOC during EEG measurements under these conditions, it was possible to isolate and remove the brain activity related to movement. As with the other three conditions, the participant was asked to press the button for the first time after the beep sound, leave at least 2 s between each button press, and then press the button at intervals of at least 5 s. This allowed for accurate subtraction. In this condition, the device is worn, but the participant is not informed that pressing the button will activate the device. Instead, the instruction is given to ‘press the button at intervals of at least 5 s,’ although the device is not triggered.

**Figure 5:**
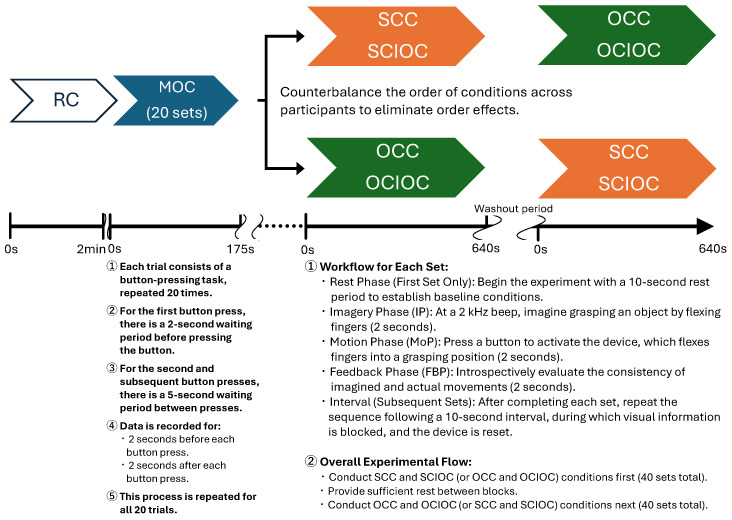
Experimental protocol timeline. The figure illustrates the temporal sequence of events during each trial, including the Imagery Phase (IP), Motion Phase (MoP), and Feedback Phase (FBP). EEG recording was continuous throughout the experiment, but data epochs were extracted specifically from the IP, MoP, and FBP intervals, spanning from 2 s before to 4 s after the button press. The timeline also includes intervals between trials for resetting the device and blocking visual information.

In relation to these conditions, the following points must be considered when determining the number of repetitions for each task: improvement of the signal-to-noise ratio (S/N ratio), consideration of within- and between-individual variability, minimization of fatigue effects, and comparability with previous research in the same field. Therefore, based on a report that verified ERD during grasping movements using EEG [52], and considering these factors, this study set all conditions to 20 repetitions. In addition, repeating a single condition increases not only the prediction of finger movement but also the anticipation of responses in the MoP phase for each condition; therefore, it is necessary to design the task to minimize such prediction. Therefore, the SCC and SCIOC were combined as self-control tasks and the OCC and OCIOC as other-control tasks, with each set performed at 40 times in total. The order of conditions for each task was randomized. In the experimental procedure, MOC was conducted first, followed by SCIOC and OCIOC, with a sufficient washout period in between. A sufficient interval was provided between the two tasks, and the order of the tasks was varied for each participant to account for order effects. For each trial, the participants followed the protocol illustrated in Figure 5.

#### 2.2.4. Evaluation of Brain Function Activity Using EEG

##### Electroencephalography

Electroencephalography data were acquired using a Polymate V AP5148 biosignal acquisition device (Miyuki Giken Co. Ltd., Tokyo, Japan). A measurement cap equipped with active dry electrodes was placed on the participants before the start of each experiment. The electrode placement sites were based on the International 10–20 System [53] and included Fpz, Fz, Cz, Pz, Oz, FP1, FP2, F7, F8, F3, F4, C3, C4, C5, C6, P3, P4, P7, P8, O1, O2, T7, T8, CPz, CP1, CP2, PO3, and PO4. The reference electrodes were placed on both earlobes, and signals were recorded at a sampling frequency of 1 kHz. In the EEG measurements, a low-pass filter (30 Hz) was applied to eliminate high-frequency noise, and a high-pass filter (8 Hz) was applied to eliminate low-frequency noise [54]. To reduce artifacts caused by eye movements and blinking, electrodes were attached below the eyes, and eye movements were recorded simultaneously. In addition, during the experiment, earplugs were attached to both ears to block the auditory stimuli for participants under all conditions.

##### EEG Data Analysis

The following additional exclusion criteria were applied during data analysis of the EEG data obtained from the Self-Controlled Task (SCC and SCIOC), Other-Controlled Task (OCC and OCIOC), and MOC: (1) technical problems due to poor electrode attachment or high impedance and (2) deviations from the experimental protocol (e.g., if the participant was unable to perform the assigned task correctly). If any of these issues were observed, the data were excluded from the analysis.

As part of the analysis procedure, the EEG data were preprocessed using EEGLAB toolbox in MATLAB (R2023b) [55]. The data were first bandpass filtered between 8 and 30 Hz using a zero-phase FIR filter. Independent component analysis (ICA) was then applied to remove artifacts such as eye blinks, eye movements, and muscle activity [56,57,58]. In addition, for the data from the 40 self-control tasks and 20 MOC tasks, the averaged MOC data were subtracted to isolate the brain nerve activity related to button-pressing movements. Next, for the EEG data that underwent the above processing, 20 datasets were extracted for each of the SCC, IOC, SCIOC, and OCIOC conditions. First, to examine brain nerve activity related to the IP and MoP, we focused on the C3 electrode site. This is because the C3 site is thought to reflect activity in the primary motor cortex (left hemisphere), which is associated with right-hand movement [59]. To convert the C3 time-domain signal into the frequency domain and analyze the frequency components, we performed a Fast Fourier Transform (FFT) [60] using a window width of 250 ms and 75% overlap, and then calculated the power value (μV^2^). Based on previous studies [34,61], we analyzed two frequency bands: the mu-wave band (8–12 Hz) and the beta-wave band (12.5–30 Hz). From this, we calculated the ERD values [34] for the mu wave (Formula (1)) and beta wave band (Formula (2)) during the IP and MoP phases of SCC, OCC, SCIOC, and OCIOC. The formulas for each ERD value (1) and (2) are as follows:

(1) μERD %=Rμ−AμRμ×100

(2) βERD %=Rβ−AβRβ×100

where R is the power value of each frequency band during rest, and A is the power value of each frequency band during the IP or MoP task phase. ERD indicates the degree to which EEG power decreases during a task compared to rest and is considered to reflect the activity level of the cerebral cortex [62]. Mu waves reflect the activity of motor-related brain regions; however, they are known to exhibit large individual differences. Conversely, β waves reflect the overall level of neural activity, and their attenuation indicates broad cortical activity related to motor control. This idea is supported by several previous studies. Formaggio et al. reported that the occurrence patterns of μERD and βERD are related to the complexity and proficiency of the movement [63], while Kranczioch et al. found they are associated with the clarity of MI [64]. Since μERD and βERD directly reflect changes in brain activity in their respective frequency bands, using these attenuation values may more specifically capture activity changes in motor-related brain regions.

Furthermore, to evaluate neural activity related to feedback processing after MoP and at the end of movement, we calculated beta-band (12.5–30 Hz) power values at the C3 electrode site during FBP and performed ERS analysis [65,66]. Equation (3) was used to calculate ERS [34], as follows:

(3) βERS %=Aβ−RβRβ×100

Rβ indicates the average power value of the resting β wave band, and Aβ indicates the average power value of the β wave band during FBP. In FBP, data for the 2 s introspection period regarding the consistency of the exercise results were used for analysis. In the ERS analysis, we focused on the feedback processing ability of FBP in the SCC, OCC, SCIOC, and OCIOC conditions. ERS in motor-related brain regions is thought to provide information complementary to ERD, particularly as an indicator of post-movement cognitive processing, motor comparison, and matching functions [34,67]. In other words, while βERD releases cortical inhibition to enable motor execution and cognitive flow, βERS is a signal of brain activity that maintains the current motor or cognitive set [68,69]. By calculating and verifying these values, it is possible to evaluate the consistency between MI and actual ME, and to quantitatively assess cognitive processing related to the end of movement.

Next, we analyzed the EEG data using the three-dimensional imaging filter eLORETA (http://www.uzh.ch/keyinst/loreta.htm, accessed on 16 August 2024) [70]. eLORETA was used to estimate the electrical patterns of the cerebral cortex from scalp potentials recorded at each electrode site. The algorithm uses specific weights in the weighted least-squares inversion method to accurately localize any focal source in the brain [70]. Based on the principles of linearity and superposition, arbitrary distributions can be localized with reasonable accuracy. The current iterative process of eLORETA uses 6239 cortical gray matter voxels with a spatial resolution of 5 mm in the MNI-based head model [71]. Currently, eLORETA is considered useful for estimating sources of neural activity in the brain and is widely used in various EEG studies [72,73]. Using eLORETA, we calculated and verified the neural activity values of the IP, MoP, and FBP under the SCC, OCC, SCIOC, and OCIOC conditions. To identify areas of dominant neural activity in each phase, statistical analysis was performed using Statistical Non-Parametric Mapping (SnPM) to identify significant activity areas. The procedure involved within-condition analysis, performing 5000 permutation tests within the eLORETA software (version 20240816, accessed on 16 August 2024) and applying the maximum statistic method to address the issue of multiple comparisons [74]. The significance level was set at *p* < 0.05 (adjusted), and brain regions showing statistically significant activity were extracted as Regions of Interest (ROIs). The output included the mean neural activity value (mean ± SD μA/mm^2^) for all voxels. Brain regions with activity values exceeding two standard deviations above the mean, corresponding to a significance level of 5%, were identified as high-activity regions. These verifications help clarify brain activity patterns during MI and grasping movements.

#### 2.2.5. Statistical Analysis

The normality of the ERD values for the mu and beta wavebands and the FBP beta waveband ERS values for each condition was confirmed using the Shapiro–Wilk test. If normality was not confirmed, an appropriate non-parametric test was used. For the ERD values of the mu and beta wavebands, a two-way repeated measures analysis of variance was conducted for the following two factors: phase condition (IP × MoP) and control condition (SCC, OCC, SCIOC, and OCIOC). For multiple comparisons, Bonferroni correction was applied, and the significance level was set at 5%. The effect size was calculated as η^2^ (eta-squared) and was interpreted in accordance with Cohen’s criteria [75]. For the ERS value in the FBP, a one-way repeated-measures analysis of variance was conducted to examine the differences between the control conditions. For multiple comparisons, Bonferroni correction was applied, and the significance level was set at 5%. The effect size was calculated as η^2^ and interpreted using Cohen’s criteria in the same way. IBM SPSS Statistics (version 29.0.0, IBM Corp.) was used for statistical analysis.

Regarding the neural activity values obtained using eLORETA in each condition, for IP, the brain regions that showed significantly higher neural activity than the grasping MI were extracted as ROIs, the neural activity values (μV/mm^2^) for these regions were calculated, and a two-way repeated-measures analysis of variance was conducted for the two factors of the four conditions (SCC, OCC, SCIOC, OCIOC) × ROIs. The significance level was set at less than 5%. For FBP, the same analysis method as that for IP was used to examine the activity of areas related to feedback processing of exercise results and the comparison and matching of exercises. In each analysis of variance, the main effects and interactions of the two conditions were examined to clarify the detailed differences between the conditions and areas. The Bonferroni method was used for post hoc testing to control type I errors due to multiple comparisons. In the MoP, comparisons were made using independent sample two-sample *t*-tests for SCC vs. OCC, SCC vs. SCIOC, OCC vs. OCIOC, and SCIOC vs. OCIOC, with the aim of examining the differences in brain function between self-regulation and other-regulation. In this case, we used the eLORETA multiple response *t*-test using the SnPM function [76], a program built into eLORETA. Although it would be preferable to use an analysis of variance, we decided to use the t-test because LORETA does not have an analysis of variance function. While analysis of variance allows for simultaneous testing of differences between each condition, the *t*-test only allows for testing of differences between two conditions, which can cause multiple comparison problems. Therefore, multiple comparisons were solved using a randomization test [70]. The significance level was set at 5%. The brain activity areas that showed significant differences were colored red or blue in the MNI brain images for the condition group with higher activity.

### 2.3. Ethical Considerations

Before the experiment, the participants were given a written and verbal explanation of the purpose and content of the research using an information sheet. Consent for participation in this study was obtained through signing a consent form. The Kyoto Tachibana University Research Ethics Committee approved this study (approval number: 22–03).

## 3. Results

Twenty-five participants were recruited initially. Among these, four were excluded for the following reasons: (1) technical problems due to poor electrode attachment or high impedance (*n* = 3) and (2) deviation from the experimental protocol (*n* = 1). Finally, data from 21 participants were analyzed. In addition, all participants scored five points for item 5Vd of the KVIQ, indicating normal motor imagery ability. We checked whether the participants were able to follow the motor imagery instructions in the IPs of the SCC and SCIOC and found that all participants succeeded.

### 3.1. ERD and ERS During MI and ME

The normality of the data distribution was confirmed using the Shapiro–Wilk test, and it was confirmed that all variables followed a normal distribution (*p* > 0.05). For the μERD, βERD, and IP/MoP values under the four conditions (SCC, OCC, SCIOC, and OCIOC), the μERD value for SCC was 35.1 ± 8.1% for IP and 14.6 ± 7.0% for βERD. For MoP, a μERD value of 49.3 ± 80.0% and a βERD value of 35.0 ± 10.9% were observed. For OCC, a μERD value of 35.6 ± 7.9% and a βERD value of 15.1 ± 7.8% were observed for IP, while a μERD value of 40.5 ± 6.4% and a βERD value 18.4 ± 6.1% were observed for MoP. For OCIOC, the IP value was 33.7 ± 7.8%, and the βERD value was 14.9 ± 9.5%. For MoP, the μERD value was 31.1 ± 7.7%, and the βERD value was 10.9 ± 6.1%. For SCIOC, the IP μERD value was 34.1 ± 6.2%, and the βERD value was 13.2 ± 8.7%. For MoP, the μERD value was 35.4 ± 6.1%, and the βERD value was 14.1 ± 5.5%.

**Figure 6:**
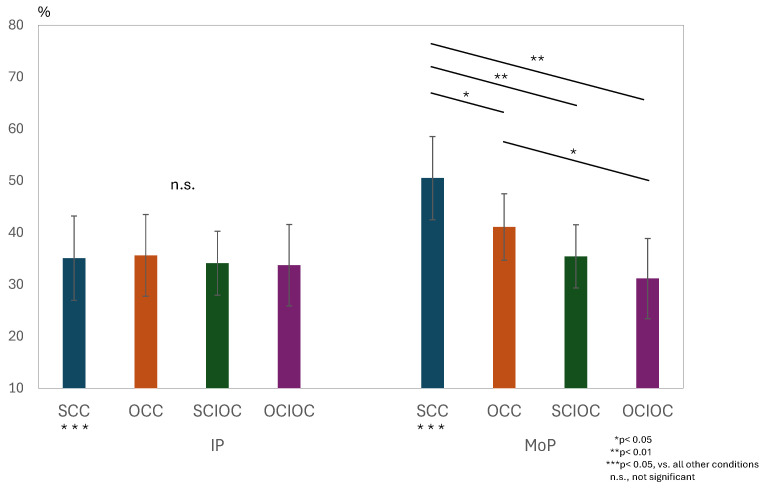
Interaction effects of phase × control conditions on μ-Band ERD values.

A two-way repeated-measures analysis of variance was conducted for the ERD values in the μ-band, with the phase condition (IP × MoP) and control condition (SCC × OCC × SCIOC × OCIOC) as factors. The results are shown in Figure 6. The interaction between the phase and control conditions was significant (F(3, 160) = 11.733, *p* < 0.001). The effect size (η^2^) of the interaction, interpreted based on Cohen’s criteria, indicated a large effect size (ηp^2^ = 0.180). The main effect of the phase condition (F(1, 160) = 18.927, *p* < 0.001, ηp^2^ = 0.106) and the main effect of the control condition (F(3, 160) = 15.973, *p* < 0.001, ηp^2^ = 0.230) were both significant. The size of the main effect for the phase condition was medium, whereas that for the control condition was large. The adjusted R^2^ value, which indicates the explanatory power of the overall model, was 0.363, meaning the model explained approximately 36.3% of the variance in the dependent variables. Next, we examined simple main effects because the interaction was significant. Multiple comparisons using the Bonferroni method revealed several significant differences among the control conditions. The SCC showed significantly higher μERD values compared to the OCC (*p* = 0.036), SCIOC (*p* < 0.001), and OCIOC (*p* < 0.001). In addition, the OCC group showed significantly higher μERD values than the OCIOC group (*p* = 0.002). However, no significant difference was observed between the SCIOC and OCIOC groups (*p* = 0.877). When the effects of the control conditions were examined for each phase condition, no significant differences were found among SCC, OCC, SCIOC, and OCIOC in IP (all *p* > 0.05). In contrast, during MoP, SCC showed significantly higher μERD values than all other conditions (all *p* < 0.05), and OCC showed significantly higher values than OCIOC (*p* < 0.05). These results indicate that the ERD values in the mu waveband were influenced by the interaction between the phase and control conditions. Specifically, the finding that SCC showed significantly higher ERD values than the other control conditions during MoP suggests that brain activity during motor planning varies depending on the degree of self-regulation.

Results of a two-way repeated-measures ANOVA (factors: Phase [IP/MoP] × Control Condition [SCC/OCC/SCIOC/OCIOC]) were obtained for μ-band ERD. A significant interaction was observed (F(3, 160) = 11.733, *p* < 0.001, ηp^2^ = 0.180). Post hoc Bonferroni tests revealed significantly higher μERD in SCC compared to OCC (*p* = 0.036), SCIOC (*p* < 0.001), and OCIOC (*p* < 0.001) during MoP. The main effects of phase (F(1, 160) = 18.927, *p* < 0.001, ηp^2^ = 0.106) and control conditions (F(3, 160) = 15.973, *p* < 0.001, ηp^2^ = 0.230) were significant. Adjusted R^2^ = 0.363. ERD: event-related desynchronization.

**Figure 7:**
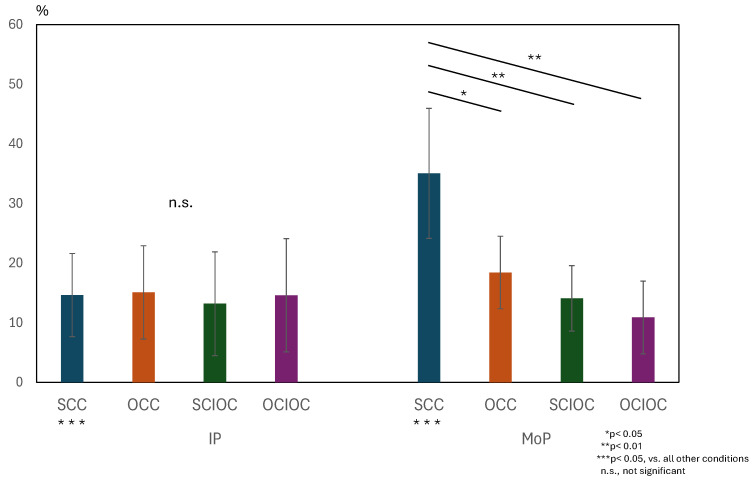
Interaction effects of phase × control conditions on β-Band ERD values.

A similar two-way repeated-measures analysis of variance was conducted for the ERD values in the beta wave band. The results are shown in Figure 7. The interaction between the phase and control conditions was significant (F(3, 160) = 18.686, *p* < 0.001). The effect size of the interaction was large (ηp^2^ = 0.259). In addition, both the main effect of the phase condition (F(1, 160) = 18.459, *p* < 0.001, ηp^2^ = 0.103) and the main effect of the control condition (F(3, 160) = 20.439, *p* < 0.001, ηp^2^ = 0.277) were both significant. The effect size for the main effect of the phase condition was moderate, whereas that of the control condition was large. The adjusted R^2^ value, which indicates the explanatory power of the overall model, was 0.435, indicating that the model explained approximately 43.5% of the variance in the dependent variable. Because the interaction was significant, we examined the simple main effects. Multiple comparisons using the Bonferroni method showed that SCC had significantly higher ERD values than the other conditions (all *p* < 0.01). The OCC group had significantly higher ERD values than the OCIOC group (*p* = 0.021). No significant differences were observed among the other conditions (all *p* > 0.05). When the effects of the control conditions were examined for each phase condition, no significant differences were found among the control conditions during IP (all *p* > 0.05). During MoP, however, SCC showed significantly higher βERD values than all other conditions (all *p* < 0.001). These results indicate that ERD values in the β band were also affected by the interaction between the phase and control conditions. In particular, the significantly higher ERD values in the β band for SCC compared to the other control conditions during MoP suggests that brain activity in the β band during motor planning also varies depending on the degree of self-regulation.

Two-way repeated-measures ANOVA (factors: Phase × Control Condition) for β-band ERD was performed. A significant interaction was found (F(3, 160) = 18.686, *p* < 0.001, ηp^2^ = 0.259). SCC showed higher βERD than all other conditions during MoP (*p* < 0.001). The main effects of phase (F(1, 160) = 18.459, *p* < 0.001, ηp^2^ = 0.103) and control conditions (F(3, 160) = 20.439, *p* < 0.001, ηp^2^ = 0.277) were significant. Adjusted R^2^ = 0.435. ERD: event-related desynchronization.

**Figure 8:**
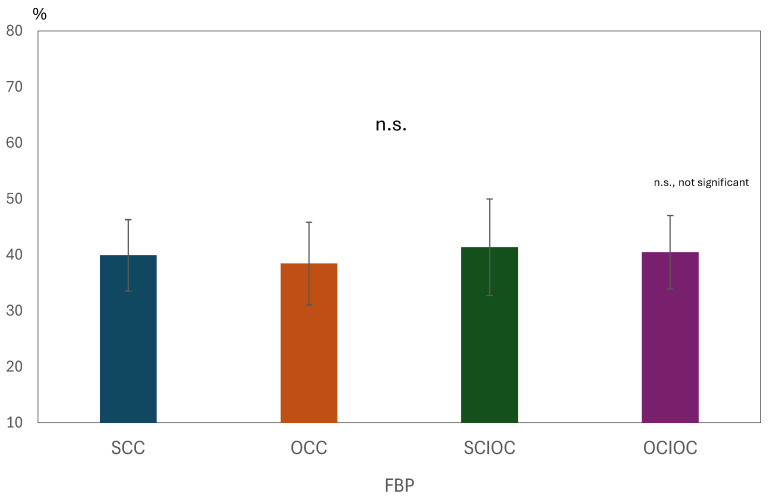
Mean β-Band ERS values during the feedback phase (FBP) for each condition.

A one-way analysis of variance was conducted on the βERS values in the FBP with the control condition as the factor. The results are shown in Figure 8. The main effect of the control condition was not significant (F(3, 80) = 0.568, *p* = 0.638, ηp^2^ = 0.021). The adjusted R^2^ value, which indicates the explanatory power of the overall model, was −0.016, indicating that the model explained virtually no variance in the dependent variable. Multiple comparisons using the Bonferroni method revealed no significant differences between any of the control conditions (all *p* = 1.000). Specifically, no statistically significant differences were found in the ERS values for SCC, OCC, SCIOC, or OCIOC. These results suggest that the ERS values in the FBP were not affected by the control conditions. In other words, the influence of varying degrees of self-control on β-band brain activity in motor-related brain regions during FBP appears to be limited.

One-way ANOVA for β-band ERS across control conditions. No significant main effect was observed (F(3, 80) = 0.568, *p* = 0.638, ηp^2^ = 0.021). Post hoc tests confirmed no differences between SCC, OCC, SCIOC, and OCIOC (*p* = 1.000). Adjusted R^2^ = −0.016, indicating no explanatory power. Error bars represent standard deviation. No significant differences were found between the conditions, resulting in similar bar heights across the graph.

### 3.2. Brain Functional Activity

**Figure 9:**
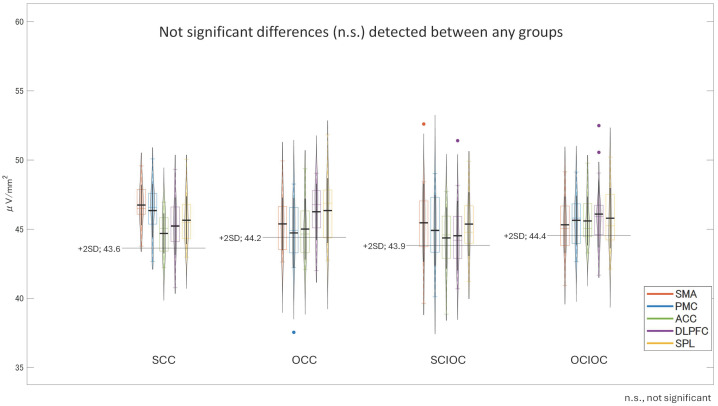
Brain region and neural activity values that showed high neural activation during IP dominance.

Using eLORETA, we calculated the brain functional activity areas and their corresponding neural activity values for the IP, MoP, and FBP across the following four conditions: SCC, OCC, SCIOC, and OCIOC. In the IP group, the brain areas that showed predominantly high neural activity were the supplementary motor area (SMA), left premotor cortex (PMC), anterior cingulate cortex (ACC), right dorsolateral prefrontal cortex (DLPFC), and left superior parietal lobule (SPL). After calculating the neural activity values for these regions and testing for the effect of participants, neither the interaction between the control and ROI conditions (F(12, 400) = 1.617, *p* = 0.084, ηp^2^ = 0.046), nor the main effects of the control condition (F(3, 400) = 3.167, *p* = 0.054) and the ROI condition (F(4, 400) = 2.207, *p* = 0.068, ηp^2^ = 0.022), were significant (Figure 9). This suggests that brain activity in the IP is not easily influenced by the degree of self-control.

Regions with high activation were SMA, left PMC, ACC, right DLPFC, and left SPL. No significant interactions (F(12, 400) = 1.617, *p* = 0.084) or main effects for control condition (*p* = 0.054) or ROIs (*p* = 0.068) were found. ηp^2^ values: interaction = 0.046; ROIs = 0.022. SMA: supplementary motor area; PMC: premotor cortex; ACC: anterior cingulate cortex; DLPFC: dorsolateral prefrontal cortex; SPL: superior parietal lobule; ROIs: regions of interest.

**Figure 10:**
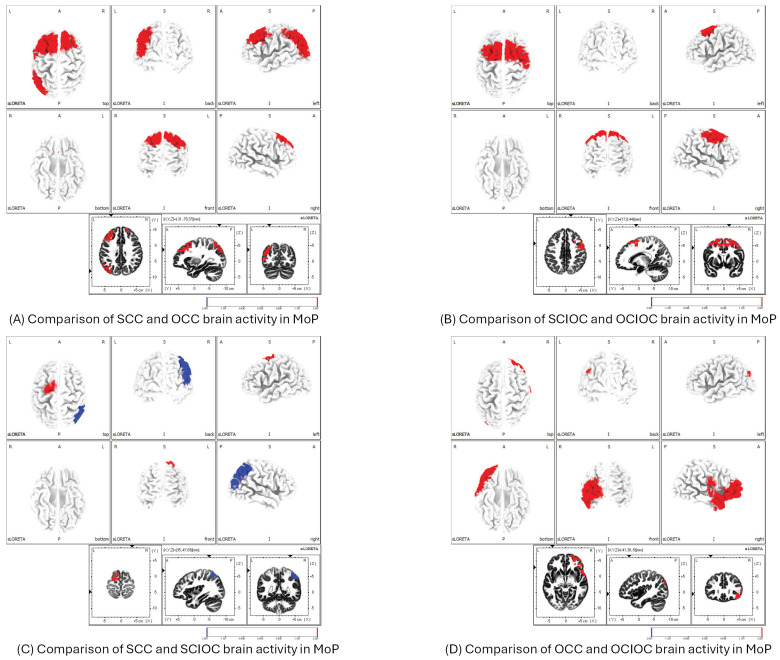
Between-condition differences in brain activation during MoP. (**A**) SCC vs. OCC: higher activation in M1, SMA, PMC, and IPL for SCC.; (**B**) SCIOC vs. OCIOC: greater activity in SMA, PMC, and dACC for SCIOC.; (**C**) SCC vs. SCIOC: SCC showed higher SMA/PMC activity; SCIOC had higher IPL activity.; (**D**) OCC vs. OCIOC: OCC exhibited elevated IPL, S2, and VLPFC activation. M1: primary motor cortex; SMA: supplementary motor area; PMC: premotor cortex; dACC: dorsal anterior cingulate cortex; IPL: inferior parietal lobule; S2: secondary somatosensory cortex; VLPFC: ventrolateral prefrontal cortex.

The MoP results showed that the SCC had significantly higher neural activity in the primary motor cortex (M1), SMA, PMC, and IPL than the OCC (Figure 10A). The comparison between SCIOC and OCIOC showed that the SCIOC group had significantly higher neural activity in the SMA, PMC, and dorsal anterior cingulate cortex (dACC), which are motor-related areas, and in brain regions involved in error detection and monitoring (Figure 10B). When SCC and SCIOC were compared, the SCC group showed significantly higher neural activity in the SMA and PMC, whereas the IPL showed significantly higher neural activity in the SCIOC group (Figure 10C). When OCC and OCIOC were compared, the OCC group showed significantly higher neural activity in the brain regions of the IPL, secondary somatosensory cortex (S2), and VLPFCS in OCC (Figure 10D). This suggests that brain activity related to processing motor feedback and planning and controlling movements increases during other-controlled movements.

**Figure 11:**
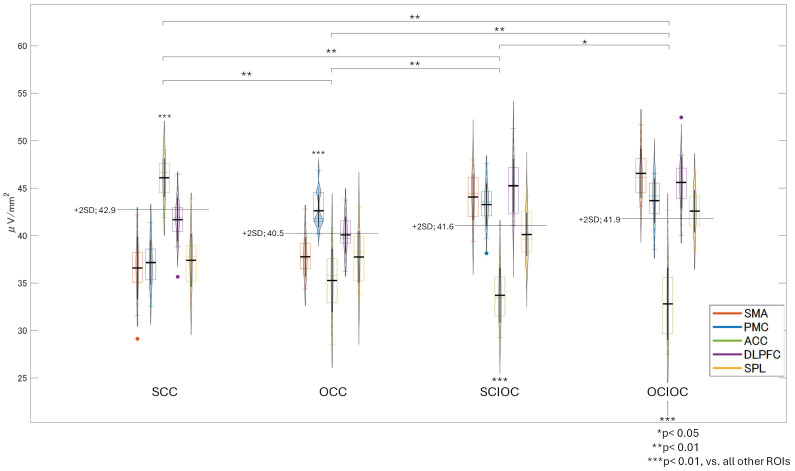
Brain regions and neural activity values that showed high neural activity during FBP dominance.

The FBP results showed that the brain regions with predominantly high neural activity varied by condition—the insula in the SCC; the IPL in OCC; the DLPFC and ACC in the SCIOC; and the ACC, DLPFC, IPL, and VLPFC in OCIOC (Figure 11). After calculating the neural activity values for these regions and conducting a test for the effect of the participants, the main effect of the control condition (F(3, 400) = 38.627, *p* < 0.001), the main effect of ROI (F(4, 400) = 70.341, *p* < 0.001), and the interaction between the control condition and ROI (F(12, 400) = 55.693, *p* < 0.001) were significant. The effect sizes were large for the main effect of the control condition (ηp^2^ = 0.225), the main effect of ROI (ηp^2^ = 0.413), and the interaction (ηp^2^ = 0.626). Bonferroni-corrected multiple comparisons revealed a complex relationship between control conditions and ROIs, with brain activity varying both across regions and by condition. The insula showed significantly lower activity compared to the ACC, IPL, DLPFC, and VLPFC (all *p* < 0.001). The ACC showed significantly higher activity than the IPL and VLPFC (*p* < 0.001), and there was no significant difference between the ACC and DLPFC. The IPL showed significantly higher activity than the DLPFC and VLPFC (*p* < 0.001). The DLPFC showed significantly higher activity than the VLPFC (*p* < 0.001). Multiple comparisons for each control condition showed that OCIOC had significantly higher activity than SCC (*p* < 0.01), OCC (*p* < 0.01), and SCIOC (*p* < 0.05) (all *p* < 0.01). SCIOC showed significantly higher activity than SCC and OCC (both *p* < 0.01). OCC showed significantly higher activity than SCC (*p* < 0.05). A detailed examination of the interaction between the control condition and ROI revealed that, in the SCC, the insula showed significantly higher activity than other brain regions (all *p* < 0.01), and no significant differences were found among the ACC, DLPFC, IPL, and VLPFC. In the OCC, the IPL showed significantly higher activity than other brain regions (*p* < 0.01), and no significant differences were found among the ACC, DLPFC, and VLPFC. In the SCIOC group, the DLPFC showed significantly higher activity than all other ROIs (all *p* < 0.01), and the ACC showed significantly higher activity than the insula and VLPFC (*p* < 0.01 for each). In the OCIOC group, the insula showed significantly lower activity compared to all other ROIs (all *p* < 0.01), and no significant differences were found among the ACC, IPL, DLPFC, and VLPFC. These results suggest that brain activity during FBP is influenced by both control conditions and ROIs, and that it changes in complex ways. In particular, the insula showed low activity under all control conditions, suggesting that it may not be involved in FBP, regardless of the degree of self-control. In contrast, the ACC and DLPFC showed relatively high activity in all control conditions, suggesting that they may function persistently regardless of the degree of self-control. The IPL and VLPFC showed significant changes in activity depending on the control condition, suggesting that they may function flexibly according to their degree of self-control.

There was a significant interaction between control condition and ROI (F(12, 400) = 55.693, *p* < 0.001, ηp^2^ = 0.626). Main effects were significant for control condition (F(3, 400) = 38.627, *p* < 0.001, ηp^2^ = 0.225) and ROI (F(4, 400) = 70.341, *p* < 0.001, ηp^2^ = 0.413), as described above. OCIOC showed higher overall activation than SCC, OCC (both *p* < 0.01), and SCIOC (*p* < 0.05). 

## 4. Discussion

In this study, we developed a hand motion-assistive device called flexEXO, which can be used by patients with reduced voluntary hand motion, and we examined the effects of self-regulated operation on brain activity. The results of ERD/ERS analysis of μ and β waves showed that the ERD values of μ and β waves were higher in the self-regulated motion conditions (SCC and SCIOC) than in the externally regulated motion conditions (OCC and OCIOC). This suggests that self-regulated movement promotes the activity of sensorimotor-related areas, such as the primary motor cortex, supplementary motor area, and premotor cortex. These results are consistent with previous research findings that self-regulated movement leads to higher activity in motor-related areas than externally regulated movement [77,78]. Self-regulated movement is known to promote the activity of brain areas related to motor intention, planning, and execution, which is supported by the results of this study. In addition, there was no significant difference in the ERD of the µ and β waves between the SCIOC and OCIOC groups. This suggests that the effect of self-control is limited when only motor imagery is used. In self-controlled movement (SCC) involving actual movement, it was thought that stronger motor-related brain activity would be induced by the coincidence of sensory feedback and motor prediction. Conversely, regarding the lack of a significant difference in the β—ERS values between conditions in FBP, it is thought that this is because the grasping action is a relatively simple movement, so the resynchronization (β-rebound) after the overall movement was almost constant regardless of the phase [28,79]. It was also speculated that the reason was that the ‘movement outcome reflection’ performed in FBP was a brief cognitive process that was not very taxing for the participants and did not produce significant cognitive differences between the various control conditions. Therefore, it was thought that by increasing the cognitive processing during FBP―not limiting it to simple reflection for a few seconds but by presenting more complex cognitive tasks or sensory feedback (e.g., visual error presentation or the illusion of tactile feedback)—the changes in βERS could be more easily differentiated between conditions.

The results of the EEG source estimation revealed that the SCC showed increased activity in the M1, SMA, and PMC compared to the OCC. These regions are associated with the planning and execution of movement [80,81]. Conversely, the OCC showed increased neural activity in the inferior parietal lobule (IPL), secondary somatosensory cortex (S2), and the ventrolateral prefrontal cortex (VLPFC). The IPL is a region involved in processing sensory feedback for movement and motor correction [82], while S2 processes tactile and proprioceptive sensations [83]. The VLPFC is associated with motor control and decision-making [84]. These results suggest that brain activity related to sensory feedback processing from external control and the cognitive evaluation of movements was more involved. From these results, it is suggested that brain activity related to motor intention, planning, and execution increases during self-controlled movements, while brain activity related to sensory feedback processing, correction, tactile and proprioceptive sensations, and motor control and decision-making is heightened in other-controlled movements. These findings suggest that self-controlled movement promotes activity in sensorimotor-related areas, which may contribute to motor learning and functional recovery. Previous studies have also reported that self-controlled movement plays a significant role in motor learning and functional recovery [85,86]. Therefore, it is believed that self-controlled movement enhances motor control accuracy and promotes motor learning by matching motor predictions with actual sensory feedback. In comparison with the imagery-only condition, neural activity in the SMA, PMC, and dACC was higher in the SCIOC condition than in the OCIOC condition. The dACC is thought to be involved in detecting and monitoring movement errors [87]. This suggests that in self-regulatory motor imagery, in addition to motor-related regions, brain activity related to error detection and monitoring is also increased.

Furthermore, a comparison of the brain activity during FBP showed that the insula was more active in SCC than in the other conditions. The insula is believed to be a brain region involved in monitoring the internal state of the body and self-awareness [88]. This suggests that self-regulatory movement increases brain activity related to the internal monitoring of the results of movement and self-awareness. Conversely, in the OCIOC condition, the ACC, DLPFC, IPL, and VLPFC activity was higher than in the other conditions. The ACC and DLPFC are brain regions involved in error detection and cognitive control [89,90], whereas the IPL and VLPFC are regions involved in processing sensory feedback and the motor control of movement, as mentioned above. These results suggest that when imagining the control of another person’s movement, brain activity related to external monitoring of the results of movement, cognitive evaluation, and feedback processing increases. The differences in activity in the insula, DLPFC, and ACC during FBP are thought to be due to different degrees of introspection, error detection, and cognitive reevaluation during feedback between the conditions. In SCC, movement execution and sensory feedback were more likely to coincide with one’s own intentions; therefore, the insula, which is involved in monitoring internal bodily states, was activated [88]. It is thought that a specific self-awareness process occurred compared to the other conditions. In contrast, in OCIOC, to monitor any inconsistencies or discomfort with external control, the ‘pathway for evaluating and correcting external input’ such as the ACC, IPL, and VLPFC, etc., was more strongly activated. It is highly likely that the cognitive interpretation of the results of movement became important.

The flexEXO developed in this study was designed using lightweight, flexible materials and can provide appropriate assistive force while following the complex movements of the fingers. It is believed that the use of such assistive devices will enable self-controlled exercise training even for patients with reduced motor function. In recent years, the development of exercise-assistive devices using soft robotics has progressed, and their effectiveness has been reported [36]. The flexEXO was developed based on the findings of these previous studies, and its effectiveness was expected. Compared with conventional rigid devices, soft robotic-based motion-assisted devices can achieve more natural movement and provide appropriate assistance in accordance with the wearer’s movement intentions. In addition, the use of flexible materials improves the wearing experience and safety and enables long-term use.

Motion-assisted devices, such as flexEXOs, have the potential to play an important role in the rehabilitation of patients with impaired motor function due to brain damage such as stroke. Previous studies have also reported on the effectiveness of rehabilitation using exercise-assisted devices for stroke patients [91,92]. The results of this study support the findings of these previous studies and suggest that flexEXOs may be effective for the rehabilitation of patients with stroke. In addition to a decline in motor function, patients with stroke often experience a decline in sensory and cognitive functions. Using flexEXOs may help promote the recovery of not only motor function but also sensory and cognitive functions. Additionally, rehabilitation using flexEXOs respects the independence of the patient and encourages active participation. This is expected to improve patient motivation and enhance the effectiveness of rehabilitation. flexEXOs can also be applied to patients with other neurological or musculoskeletal disorders that have resulted in a decline in hand motor function, not just in stroke patients. For example, patients with spinal cord injuries or neuromuscular diseases often have difficulty performing voluntary finger movements. Using flexEXOs, it is possible to provide these patients with self-regulated motor training, which is hoped to help maintain and improve their remaining motor functions. Previous studies have also reported the effectiveness of rehabilitation using exercise-assisted devices for patients with spinal cord injuries or neuromuscular diseases [93,94]. Patients with spinal cord injuries and neuromuscular diseases often experience severe motor function decline, and conventional rehabilitation may not be effective enough. Using flexEXOs, it may be possible to provide effective rehabilitation for these patients. Furthermore, flexEXOs can also be applied in fields other than rehabilitation. For example, it could be used to maintain and improve motor function in the elderly or to improve manual dexterity in occupational therapy. In addition, it may be possible to achieve more effective motor learning by combining it with training systems that use VR or AR.

The results of this study suggest the importance of self-regulated movement in the development of movement-assistive devices. Further research is needed to apply flexEXOs and other movement-assistive devices in clinical settings. In particular, it is necessary to verify the effectiveness of flexEXOs by conducting research on actual patients such as those with stroke. In addition, the effects of long-term use and the effects of combining flexEXOs with other rehabilitation techniques also need to be examined. There are also issues to be addressed in the clinical application of movement-assistive devices, such as their safety, durability, and cost. To resolve these issues, it is necessary to promote research in collaboration with experts in fields such as engineering, medicine, and rehabilitation science.

One limitation of this research is that it was conducted on healthy participants; therefore, it is not clear whether the results can be applied to actual patients. In addition, as the experiment was conducted on a relatively small sample size, care must be taken when generalizing the results. We acknowledge that the exclusion of female participants and the small sample size of healthy young males with low age variation are limitations of our study. These factors may limit the generalizability of our findings to clinical populations, such as stroke patients. Future studies should investigate the use of the flexEXO in larger, more diverse samples, including both male and female participants and clinical populations, to validate its potential for neurorehabilitation and to confirm and extend our results. In the future, it will be necessary to conduct research on actual patients, such as those with stroke, and verify the effectiveness of flexEXOs. Furthermore, it will also be necessary to consider the effects of long-term use and the combination of it with other rehabilitation techniques. In addition, this study did not directly examine the effects of exercise autonomy, contingency, or learning. However, previous studies have reported that exercise autonomy and contingency affect exercise performance and learning effects [95,96,97], so it is possible that self-regulated exercise may promote more effective motor learning by increasing exercise autonomy and contingency. Another limitation of this study is the simplified feedback phase task design, with a 2 s reflection period. While this duration was chosen to maintain a reasonable pace for the experiment, it may not fully capture the nuanced cognitive differences between conditions. Future studies could investigate the impact of different feedback intervals on the cognitive processes involved in self-controlled and externally controlled movements. Incorporating varying reflection periods or the inclusion of specific feedback prompts may provide additional insights into the optimal feedback design for neurorehabilitation using flexEXOs. While our study did not directly compare flexEXOs to other devices, the observed differences in brain activity patterns between self-controlled and externally controlled conditions suggest that flexEXOs may have the potential to promote more effective motor learning and neurorehabilitation. However, future research should directly compare the effectiveness of flexEXOs with other technologies to establish their efficiency and potential benefits. In the future, it will be necessary to verify the effects of flexEXOs from these perspectives as well.

The results of this study suggest that self-controlled exercise promotes activity in sensorimotor-related areas and may contribute to motor learning and functional recovery. It is believed that the use of exercise-assisted devices such as flexEXOs will enable patients with impaired motor function to undergo self-controlled exercise training. The results of this study have the potential to lead to innovations in the field of rehabilitation, and we look forward to future developments. The development of exercise-assisted devices requires cooperation among experts in fields such as engineering, medicine, and rehabilitation science. In addition, issues such as safety, durability, and cost must be addressed in order to apply the developed devices in clinical practice. By resolving these issues and widely disseminating exercise-assisted devices, we expect that we will be able to contribute to improving the quality of life for patients with impaired motor function.

## 5. Conclusions

In this study, we developed a hand-motion assist device, flexEXO, that can be used by patients with reduced voluntary hand movement and examined the effects of self-regulated operation on brain activity. The results of ERD/ERS analysis of μ- and β-waves and source estimation of EEG signals suggested that self-regulated tasks may promote activity in motor-related areas. In particular, significant activity was observed in brain regions related to the planning and execution of movements, such as the M1, SMA, and PMC. Conversely, in the case of an externally controlled task, such as one guided by another person, it was shown that activity in brain regions related to the processing and correction of sensory feedback of movement, such as the SPL, increased. flexEXO is designed using lightweight and flexible materials and can provide appropriate assistive force while following complex finger movements. It is believed that the use of such assistive devices will enable patients with reduced motor function to undergo self-controlled motor training. The results of this study have the potential to play an important role in the rehabilitation of patients with reduced hand motor function due to stroke, spinal cord injury, or neuromuscular disease. The results of this study also revealed the impact of consistency between motor intention and actual movement on motor-related brain activity.

The results showed that when intention and actual movement were in agreement, there was greater activity in sensorimotor-related areas, whereas when there was no agreement, greater activity was observed in brain areas related to error detection, conflict monitoring, and higher-order cognitive functions. These findings are important for understanding the mechanisms underlying motor adaptation and learning. In the future, it will be necessary to conduct research on actual patients, such as those with stroke, to verify the effectiveness of the flexEXOs. In addition, it is necessary to investigate the effects of long-term use and the effects of combining flexEXOs with other rehabilitation techniques. Furthermore, it is necessary to clarify the effects of self-regulated exercise training using flexEXOs on the sense of agency, contingency, and exercise-related learning outcomes.

## Figures and Tables

**Table 1:** Participant characteristics (*n* = 25).

Characteristic	Mean ± SD
Age (years)	22.9 ± 2.0
Height (cm)	170.4 ± 3.8
Weight (kg)	63.2 ± 6.7
Dominant hand (Right/Left)	25/0

## Data Availability

The datasets generated and analyzed during the current study contained sensitive personal information derived from individual brainwave recordings. Due to privacy concerns and ethical considerations, these data are not publicly available. However, the corresponding author may make the datasets available upon reasonable request and subject to appropriate data protection agreements.

## Abbreviations

The following abbreviations are used in this manuscript:

Abbreviation	Definition
MI	Motor Imagery
ME	Motor Execution
SCC	Self-Controlled Condition
OCC	Other-Controlled Condition
SCIOC	Self-Controlled Imagery-Only Condition
OCIOC	Other-Controlled Imagery-Only Condition
MOC	Motion-Only Condition
MoP	Motion Phase
IP	Imagery Phase
FBP	Feedback Phase
ERD	Event-Related Desynchronization
ERS	Event-Related Synchronization
EEG	Electroencephalography
M1	Primary Motor Cortex
SMA	Supplementary Motor Area
PMC	Premotor Cortex
IPL	Inferior Parietal Lobule
S2	Secondary Somatosensory Cortex
VLPFC	Ventrolateral Prefrontal Cortex
KVIQ	Kinesthetic and Visual Imagery Questionnaire
S/N	Signal-to-Noise
flexEXO	Flexible Exoskeleton
